# The role of TolA, TolB, and TolR in cell morphology, OMVs production, and virulence of *Salmonella* Choleraesuis

**DOI:** 10.1186/s13568-022-01347-4

**Published:** 2022-01-25

**Authors:** Quan Li, Zheng Li, Xia Fei, Yichen Tian, Guodong Zhou, Yuhan Hu, Shifeng Wang, Huoying Shi

**Affiliations:** 1 College of Veterinary Medicine, Yangzhou University, Yangzhou, 225009 Jiangsu People’s Republic of China; 2 Jiangsu Co-Innovation Center for the Prevention and Control of Important Animal Infectious Diseases and Zoonoses, Yangzhou, 225009 Jiangsu China; 3 Department of Infectious Diseases and Immunology, College of Veterinary Medicine, University of Florida, Gainesville, FL 32611-0880 USA; 4 Joint International Research Laboratory of Agriculture & Agri-Product Safety (JIRLAAPS), Yangzhou University, Yangzhou, 225009 Jiangsu China

**Keywords:** Tol–Pal system, *tolA*, *tolB*, *tolR*, *Salmonella* Choleraesuis, Outer membrane vesicles, Virulence

## Abstract

The Tol–Pal system of Gram-negative bacteria is necessary for maintaining outer membrane integrity. It is a multiprotein complex of five envelope proteins, TolQ, TolR, TolA, TolB, and Pal. These proteins were first investigated in *E. coli*, and subsequently been identified in many other bacterial genera. However, the function of the Tol–Pal system in *Salmonella* Choleraesuis pathogenesis is still unclear. Here, we reported the role of three of these proteins in the phenotype and biology of *S.* Choleraesuis. We found that mutations in *tolA*, *tolB*, and *tolR* caused severe damage to the cell wall, which was supported by observing the microstructure of spherical forms, long chains, flagella defects, and membrane blebbing. We confirmed that all the mutants significantly decreased *S.* Choleraesuis survival when exposed to sodium deoxycholate and exhibited a high sensitivity to vancomycin, which may be explained by the disruption of envelope integrity. In addition, *tolA*, *tolB*, and *tolR* mutants displayed attenuated virulence in a mouse infection model. This could be interpreted as a series of defective phenotypes in the mutants, such as severe defects in envelope integrity, growth, and motility. Further investigation showed that all the genes participate in outer membrane vesicles (OMVs) biogenesis. Interestingly, immunization with OMVs from Δ*tolB* efficiently enhanced murine viability in contrast to OMVs from the wild-type *S.* Choleraesuis, suggesting its potential use in vaccination strategies. Collectively, this study provides an insight into the biological role of the *S.* Choleraesuis Tol–Pal system.

## Introduction

*Salmonella enteric*a serovar Choleraesuis (*S*. Choleraesuis), a Gram-negative bacterium, is an important swine pathogen that cause a series of severe diseases, including meningitis, hepatitis, pneumonia, and other systemic diseases (Reed et al. 1986). Moreover, it's a major zoonotic agent that could be occasionally isolated from humans and triggers huge economic damage in the porcine sector across the globe (Allison et al. 1969; Bangtrakulnonth et al. 2004; Gray et al. 1995). Thus far, the pathogenesis of *S*. Choleraesuis infections is still not fully understood (Chiu et al. 2004). Hence, it's imperative to elucidate the pathogenic mechanism of *S*. Choleraesuis.

The Tol–Pal system of Gram-negative bacteria is a multiprotein composite traversing the inner membrane, periplasm, and outer membrane (OM) (Hirakawa et al. 2020). It comprises five envelope proteins, corresponding to TolQ, TolR, TolA, TolB, and Pal (Henry et al. 2004). Three inner membrane proteins TolQ, TolA, and TolR exhibit interaction with each other through their trans-membraneous domains (Derouiche et al. 1995). Pal is an OM anchored protein interacting with the periplasmic protein TolB (Ray et al. 2000). The system plays numerous biologic functions in Gram-negative bacteria, including cell morphology, sensitivity to bile salts, and bacterial virulence (Dubuisson et al. 2005; Lahiri et al. 2011; Paterson et al. 2009). Furthermore, it has also been displayed that inactivation of the *tol*–*pal* genes negatively impacts the outer membrane integrity, resulting in increased formation of outer membrane vesicles (OMVs). OMVs primarily comprise phosphatides, periplasm and OM proteins in Gram-negative microbes, and the Tol–Pal system proteins are the essential components of the OMVs. The Tol–Pal system was 1st characterized in *E. coli* (Webster 1991), and subsequently been reported in many other bacterial genera, including *Pseudomonas aeruginosa* (Dennis et al. 1996), *Vibrio cholerae* (Heilpern and Waldor 2000), *Pseudomonas putida* (Llamas et al. 2000), *Salmonella* Typhimurium (Prouty et al. 2002), *Erwinia chrysanthemi* (Dubuisson et al. 2005), and *Salmonella* Typhi (Lahiri et al. 2011). Although some progress has been made in the research of other *Salmonella enterica* serovars, the function of the Tol–Pal system in *S*. Choleraesuis has not been documented.

Of particular note, data obtained from *S*. Typhimurium or *S*. Typhi (less from other bacteria) may not be inferred directly to *S*. Choleraesuis without experimental results (Nevermann et al. 2019; Urrutia et al. 2014). Compared with *S*. Typhimurium and *S*. Typhi, *S*. Choleraesuis behaves obvious differences in terms of disease progression and host range. They are differences in pathogenic mechanisms, probably due to the different molecular functions of some proteins. Lahiri et al. have demonstrated that there is a considerable difference in the sequence of *tolA* between *S*. Typhi and *S*. Typhimurium (Lahiri et al. 2011). Deletion of *tolA* of the two serovars exhibits entirely different phenotypes, including membrane organization, detergent resistance, and cell morphology. Nevermann et al. showed that the *tolR* mutation of *S*. Typhimurium and *S*. Typhi also presents fully differently regarding sensitivity to vancomycin, motility, and OMVs production (Nevermann et al. 2019). These results indicated that an experimental approach has to be implemented to better elucidate the function of Tol–Pal system of *S*. Choleraesuis.

In this study, and with the aim to explore the roles of *tolA*, *tolB*, and *tolR* genes of the Tol–Pal system in *S.* Choleraesuis, we constructed *tolA*, *tolB*, and *tolR* mutants via homologous recombination. Identifying these genes will help us better comprehend the additional roles of the Tol–Pal system that are difficult to observe in other bacterial genera. We found that all these genes are involved in cell morphology, membrane integrity, cell growth, motility, virulence, and OMVs biogenesis. In addition, we also described the immune responses and protective efficacy of *S.* Choleraesuis OMVs in a mouse model. In general, this study expanded our understanding of the biological role of the *S.* Choleraesuis Tol–Pal system.

## Materials and methods

### Plasmids, strains, and growth conditions

Plasmid pRE112 and *E. coli* strain χ7213 were kindly offered by Dr. Roy Curtiss III. *S.* Choleraesuis strain C78-3 (CVCC79103) were bought from China Institute of Veterinary Drugs Control. *E. coli* χ7213 and *S.* Choleraesuis strains were grown on LB agar plates or in LB broth (OXOID). When required, 25 µg/ml chloromycetin (Cm) or 50 µg/ml diaminopimelic acid was supplemented into the LB media. Plasmids and strains used in this study are presented in Table 1.

**Table 1:** Characteristics of the bacterial strains and plasmids used in this study

Strains or plasmids	General characteristics^a^	Sources or references
Bacterial strains		
C78-3	Wild type, virulent, CVCC79103	Ji et al. (2015)
*∆tolA*	Isogenic *tolA* mutant of strain C78-3	This study
*∆tolB*	Isogenic *tolB* mutant of strain C78-3	This study
*∆tolR*	Isogenic *tolR* mutant of strain C78-3	This study
χ7213	*thi‑1 thr‑1 leuB6 fhuA21 lacY1 glnV44 asdA4 recA1 RP4 2‑Tc::Mu pir*	Roland et al. (1999)
Plasmids		
pRE112	*sacB mobRP4 R6 K oriV oriT*; suicide vector; Cm^r^	Edwards et al. (1998)
pRE112-*tolA*	Suicide vector for *∆tolA*; pRE112 derivative; Cm^r^	This study
pRE112-*tolB*	Suicide vector for *∆tolB*; pRE112 derivative; Cm^r^	This study
pRE112-*tolR*	Suicide vector for *∆tolR*; pRE112 derivative; Cm^r^	This study

^a^Cm^r^: chloramphenicol resistance

### Ethics statement

Female BALB/c mice (6-week-old) were bought from the Comparative Medicine Center of Yangzhou University. The entire murine studies were completed at Yangzhou University and approved by the Administrative Committee for Laboratory Animals of Jiangsu Province [permission number SCXK (SU) 2017-0007]. The process complied with the protocols of Jiangsu Laboratory Animal Welfare and Ethical guidelines, and all endeavors were performed for the purpose of minimizing the pain of the mice.

### Construction of the *tolA*, *tolB*, and *tolR* mutants

Three mutations Δ*tolA*, Δ*tolB*, and Δ*tolR* were applied for *S.* Choleraesuis strain C78-3 using correspondent suicide vectors. The primers used for PCR amplification of DNA fragments corresponding to the upstream and downstream flanking regions of the *tolA*, *tolB*, and *tolR* genes are listed in Table 2. In brief, the upstream flanking regions (L) and downstream flanking regions (R) of the target genes were fused as complete fragments (LR) via overlapping PCR, and then cloned into pRE112 via the SacI and KpnI restriction sites. The mutations were constructed in C78-3 by conjugating with χ7213 carrying suicide plasmids as previously reported (Curtiss et al. 2009; Roland et al. 1999). PCR confirmation of the deletions using two primer sets of flanking regions (A/D) and internal regions (E/F), and sequencing by Tsingke Biotechnology Co., Ltd. (Beijing, China).

**Table 2:** Primers used for PCR amplification and detection

Primers	Sequences (5′–3′)^a^	Function	Length (bp)	Restriction enzyme
*tolA*-A	CGCA**GAGCTC**ATTATTGAGGTTTCCGGAGTA	Upstream flanking regions of *tolA*	301	SacI
*tolA*-B	TCTCGGTTCCCAAAAAACTGT			
*tolA*-C	ACAGTTTTTTGGGAACCGAGAATACTTTTCTTTATGGAAGTT	Downstream flanking regions of *tolA*	322	
*tolA*-D	CGG**GGTACC**TACCGCTATTGCGTAAATCTG			KpnI
*tolA*-E	AGGAGCGGTTGAAACAACTTG	Internal regions of *tolA*	562	
*tolA*-F	CTGAGATCGCCAAGCAGATCG			
*tolB*-A	CGCA**GAGCTC**AATGTGTCTTGCATATTAGCCTG	Upstream flanking regions of *tolB*	305	SacI
*tolB*-B	CATATCTCCCATACCTGGGCCTG			
*tolB*-C	CAGGCCCAGGTATGGGAGATATGTAATAATTAATTGATTACTAA	Downstream flanking regions of *tolB*	269	
*tolB*-D	CGG**GGTACC**ACTTGTCGAGATCGAAGTAAA			*Kpn* I
*tolB*-E	TGCGTTATGCAGGTCATACCG	Internal regions of *tolB*	415	
*tolB*-F	TGACCGGAGGCGAGATCCATA			
*tolR*-A	CGCA**GAGCTC**CGTTTCTTGGCACGGTAGGCT	Upstream flanking regions of *tolR*	314	SacI
*tolR*-B	GGCTTACCCCTTGTTGCTTTC			
*tolR*-C	GAAAGCAACAAGGGGTAAGCCAGTCTGCGTCCCGTTGGCTTG	Downstream flanking regions of *tolR*	320	
*tolR*-D	CGG**GGTACC**GTTGCAGCTTTTTACGCTCTT			KpnI
*tolR*-E	AGGTCGTCGCGAACTTAAGTC	Internal regions of *tolR*	351	
*tolR*-F	AGCGCTTTAATTATTTCATCG			

^a^Bold nucleotides denote enzyme restriction sites

### Transmission electron microscopy (TEM)

TEM analysis was completed to investigate the role of *tolA*, *tolB*, and *tolR* on the morphology of *S.* Choleraesuis as previously described with minor modification (Elhenawy et al. 2016). In brief, bacterial strains were cultivated in LB liquid medium with shaking at 37 °C and collected at the mid-exponential phase (OD_600_ = 0.9). Afterwards, the cells were allowed to absorb onto carbon-coated copper grids and negatively stained with 1% uranyl acetate. The samples were allowed to air dry and examined with a Tecnai T12 transmission electron microscope.

### Analysis of resistance to sodium deoxycholate and vancomycin

Assays for resistance to deoxycholic acid were performed as previously described (Nevermann et al. 2019). Briefly, bacterial strains were grown in LB to an OD_600_ of 0.9 and harvested via centrifugating. Then, the cells were cleaned two times with PBS and subjected to resuspension in 0.5% sodium deoxycholate or PBS at 37 °C for 2 h. Microbial survival was counted following plating serial dilutions onto LB agar. The survival rate was computed as (CFU in deoxycholic acid/CFU in PBS) × 100%. The antibiotic sensitivity assay of vancomycin was performed as previously described (Li et al. 2019), using Kirby-Bauer disc diffusion technique. The vancomycin disks contained 30 µg of the antibiotic. Every experiment was finished in 3 independently performed biology duplicates.

### Bacterial growth curve assays

The *S.* Choleraesuis C78-3 and its mutants (Δ*tolA*, Δ*tolB*, and Δ*tolR*) were cultivated in LB to an OD_600_ of 0.9 and added to 50 ml LB broth (1:200). The cultures were cultivated by vigorous shaking (200 rpm) at 37 °C for 12 h. The OD_600_ of C78-3 and three mutants were quantified at 60 min interval via a spectral photometer (Bio-Rad). Meanwhile, bacteria numbers were counted every hour following plating serial dilutions onto LB agar. Every experiment was finished in 3 independently conducted biology duplicates.

### Motility assays

Motility assays were performed according to a previously described method with minor modification (Morgan et al. 2014). In short, bacterial strains were cultured in LB to an OD_600_ of 0.9, and then diluted 1:10 in fresh LB and 1 µl was inoculated on semi-solid (0.5%) LB agar plates containing 0.02% arabinose. The plates were cultivated for 5 h at 37 °C and cell motility was assessed via the diameter of growth halo (mm). Each assay was performed in triplicate and repeated in 3 independent replicates.

### Assessment of LD_50_ via a mouse model

To investigate the effect of inactivating *tolA*, *tolB*, and *tolR* on the virulence of *S.* Choleraesuis, the LD_50_ of C78-3 and three mutants was tested by intraperitoneal challenges with a mouse model. Briefly, bacterial strains were cultivated in LB to an OD_600_ of 0.9 and washed twice with PBS. Four groups of BALB/c mice (n = 4) were subjected to injection with the doses of 3, 3 × 10^1^, 3 × 10^2^, and 3 × 10^3^ CFU/mouse in 100 µl PBS of wild-type strain C78-3. Meanwhile, 12 groups of mice (n = 4) were subjected to injection with the doses of 5 × 10^3^, 5 × 10^4^, 5 × 10^5^, and 5 × 10^6^ CFU/mouse in 100 µl PBS of Δ*tolA*, Δ*tolB*, or Δ*tolR*, respectively. The animals with the corresponding infection were supervised daily for 30 days. LD_50_ was determined by the approach of Reed and Muench (1938).

### Purification and quantification of OMVs

Outer membrane vesicles (OMVs) of *S.* Choleraesuis C78-3 and its mutants were isolated as previously described (Muralinath et al. 2011). In brief, bacterial strains were cultivated in LB liquid medium (300 ml) at 37 °C overnight (OD_600_ = 2.1) and harvested by centrifugation (12,000×*g*, 10 min). Subsequently, the supernatants were treated with filtration by 0.45 µm sterile filtering device (Millipore, USA). OMVs were collected from the supernatant by ultracentrifugation (150,000×*g*, 3 h, 4 °C) and washed once with PBS. OMVs were then purified by density gradient centrifugation (150,000×*g*, 12 h, 4 °C) on a discontinuous gradient from 20 to 45% of Optiprep (Axis-Shield). OMVs fractions were pooled and ultracentrifuged again. The vesicles were resuspended in 2 ml PBS and stored at − 80 °C. The obtained OMVs isolation was analyzed by TEM as previously described (Nevermann et al. 2019). The yield of OMVs from *S.* Choleraesuis C78-3 and its mutants was evaluated by the protein concentration in the OMVs. Quantification of the OMVs concentration was quantified via a BCA protein analysis kit. All OMVs samples from the strains were subjected to purification and quantification at least 3 times. Each OMVs sample (8 µl) was separated by 12% SDS-PAGE, and then the protein profiles of OMVs were visualized using Coomassie Brilliant Blue R-250.

### Immunization and challenge of mice

Five groups of BALB/c mice (6-week-old, n = 5) were immunized with 100 µl PBS involving 10 µg OMVs via the intraperitoneal route. Intraperitoneal immunizations of 100 µl PBS was the negative controls. Booster immunizations were administered 3 weeks posterior to the primary immunization. Blood specimens were harvested 5 weeks posterior to the initial immunization via orbital sinus puncture. Serum IgG were evaluated by ELISA as previously described (Li et al. 2017). Two weeks after the booster immunizations, the animals were treated with 3 × 10^6^ CFU (nearly 100 × LD_50_) of the wild-type C78-3 in 20 µl PBS via the oral route. The infected mice were supervised every day for 30 days. The protection assays were finished two times, and the results were integrated for analysis.

### Statistical analysis

The numerical results were assayed via GraphPad Prism (GraphPad Prism 5, GraphPad Software, USA). Unpaired two-tailed Student’s *t*-test was employed to evaluate statistic significance. Differences were considered as significant at *P* < 0.05. All results were obtained from at least 3 independent replicates, and values were expressed as mean ± SEM.

## Results

### Construction and confirmation of the *tolA*, *tolB*, and *tolR* knockout mutants

**Fig. 1:**
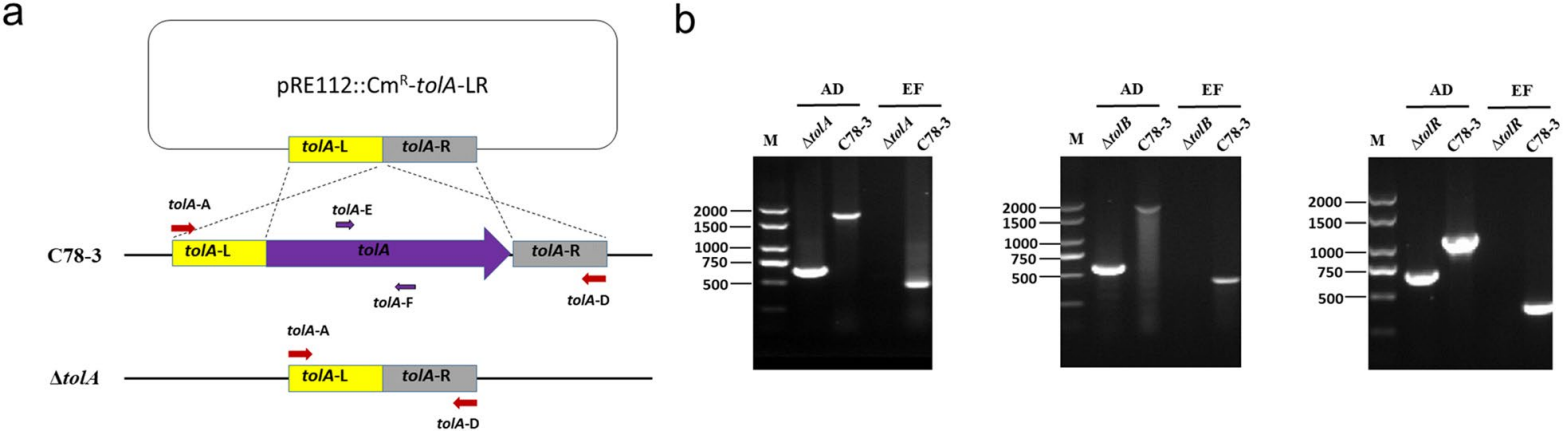
Construction and confirmation of the *tolA*, *tolB*, and *tolR* knockout mutants. **a** Schematic diagram for the generation of the *tolA*, *tolB*, and *tolR* mutants. **b** The *tolA*, *tolB*, and *tolR* mutants were verified through combined PCR. PCR confirmation of the deletions using two primer sets of flanking regions (A/D) and internal regions (E/F)

To probe the roles of Tol–Pal system in *S.* Choleraesuis, three mutants of *tolA*, *tolB*, and *tolR* were constructed via homologous recombination. A schematic representation of the homologous recombination strategy is shown (Fig. 1a). The generation of the *tolA*, *tolB*, and *tolR* mutants using a mediator based on the suicide vector pRE112. The *tolA*, *tolB*, and *tolR* mutants were verified via integrated PCR analysis using two pairs of primers, as well as sequencing. As presented in Fig. 1b, there were no fragments of Δ*tolA*, Δ*tolB*, and Δ*tolR* using inner primers (E/F), while the flanking primers (A/D) amplified smaller fragments from the mutants in contrast to those amplified from the parental strain C78-3. These results showed that Δ*tolA*, Δ*tolB*, and Δ*tolR* were constructed successfully.

### Morphological characterization of *S.* Choleraesuis C78-3 and three mutants

**Fig. 2:**
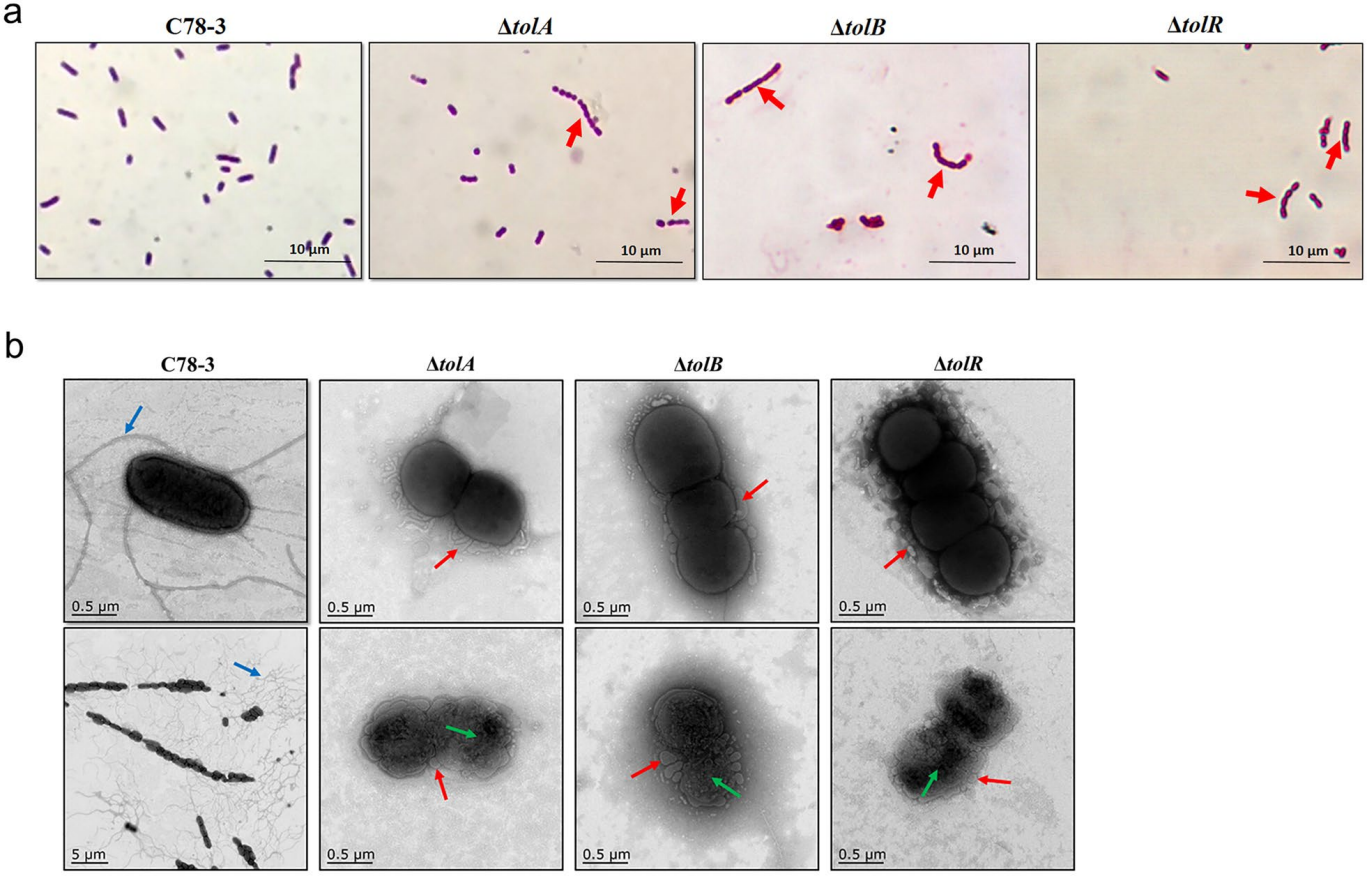
Morphological characterization of *S.* Choleraesuis C78-3 and three mutants. **a** Gram staining of C78-3 and three mutants grown in LB were examined by light microscopy (×1000). The Δ*tolA*, Δ*tolB*, and Δ*tolR* strains grew in chains of coccobacilli (red arrow). **b** Morphology characteristics of C78-3 and three mutants were evaluated by TEM. C78-3 was rod-shaped and presented long flagella (blue arrow). The Δ*tolA*, Δ*tolB*, and Δ*tolR* strains showed an altered morphology with long chains and formed vesicles at the cell surface (red arrow), while some mutants were severely damaged in their cell morphology (green arrow)

Considering that the Tol–Pal system of Gram-negative bacteria toward maintaining outer membrane stability, we speculated that deletion of *tolA*, *tolB*, and *tolR* might influence the phenotypes of *S.* Choleraesuis. Cell morphology of C78-3 and three mutants were examined by light microscopy after Gram staining. In LB broth, the wild-type *S.* Choleraesuis C78-3 grew as single rods (Fig. 2a). Under the same conditions, Δ*tolA*, Δ*tolB*, and Δ*tolR* grew in chains (3 to 10 cells) of coccobacilli (Fig. 2a) (red arrow). In order to further confirm the results, we evaluated the cell morphology by TEM. C78-3 was rod-shaped with the average size of 1.6 × 0.7 µm and presented long flagella (blue arrow). In contrast, Δ*tolA*, Δ*tolB*, and Δ*tolR* presented an altered morphology with spherical forms and long chains, which is consistent with the above observations (Fig. 2b). Of particular note, the cell morphology among the Δ*tolA*, Δ*tolB*, and Δ*tolR* strains are very similar. The three mutants formed vesicles at the cell surface (red arrow), but had no flagella. We also found that some mutants were severely damaged in their cell morphology (green arrow). Therefore, *S.* Choleraesuis *tolA*, *tolB*, and *tolR* genes participate in the maintenance of cell morphology.

### Characterization of Δ*tolA*, Δ*tolB*, and Δ*tolR* regarding the envelope integrity

**Fig. 3:**
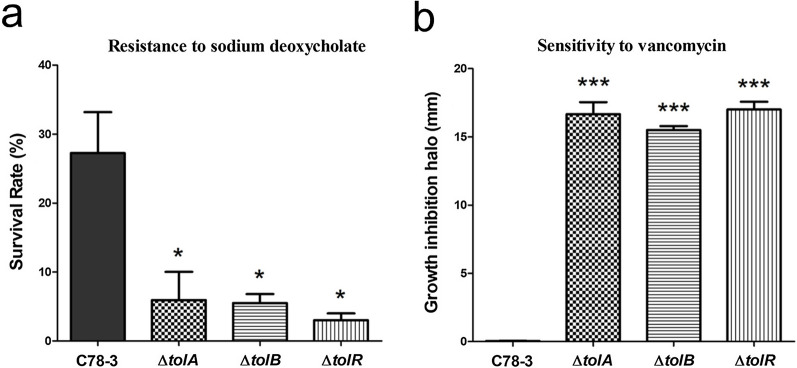
Resistance to sodium deoxycholate and vancomycin of C78-3 and three mutants. **a** Survival of C78-3, Δ*tolA*, Δ*tolB*, and Δ*tolR* in 0.5% sodium deoxycholate. Bacterial strains were cultivated in LB to an OD_600_ of 0.9. Then, the cells were resuspended in 0.5% sodium deoxycholate or PBS at 37 °C for 2 h. The survival rate was computed as (CFU in deoxycholic acid/CFU in PBS) × 100%. **b** Resistance to vancomycin of C78-3, Δ*tolA*, Δ*tolB*, and Δ*tolR* strains. The vancomycin disks contained 30 µg of the antibiotic. Each assay was completed in 3 independently conducted biology duplicates. *P < 0.05; **P < 0.01; ***P < 0.001

Previous studies found that the *tol–pal* genes of *E. coli* are important in maintaining outer membrane integrity (Lazzaroni et al. 1999). This phenomenon was also reported in *S.* Typhimurium (Paterson et al. 2009) and *Erwinia chrysanthemi* (Dubuisson et al. 2005). To determine whether deletion of *tolA*, *tolB*, and *tolR* affect the envelope integrity of *S.* Choleraesuis, assays for resistance to deoxycholic acid were performed. All mutants were more susceptible to 0.5% sodium deoxycholate in contrast to that of the parental strain (Fig. 3a). To further probe the envelope integrity, the sensitivity of Δ*tolA*, Δ*tolB*, and Δ*tolR* towards vancomycin were analyzed. In essence, Gram-negative bacteria exhibit resistance to vancomycin due to the limit of diffusible molecules through the microbial envelope (Pimenta et al. 1999). Therefore, the increase in sensitivity to vancomycin can be explained by the increase in permeability. Our results showed that wild-type *S.* Choleraesuis presented full resistance to vancomycin. In contrast, all the mutants revealed complete sensitivity to vancomycin (Fig. 3b). The *tolA*, *tolB*, and *tolR* mutants exhibited increased susceptibility to sodium deoxycholate and vancomycin, indicating that the envelope integrity might be damaged in these cases.

### The roles of *tolA*, *tolB*, and *tolR* in cell growth, motility, and virulence of *S.* Choleraesuis

**Fig. 4:**
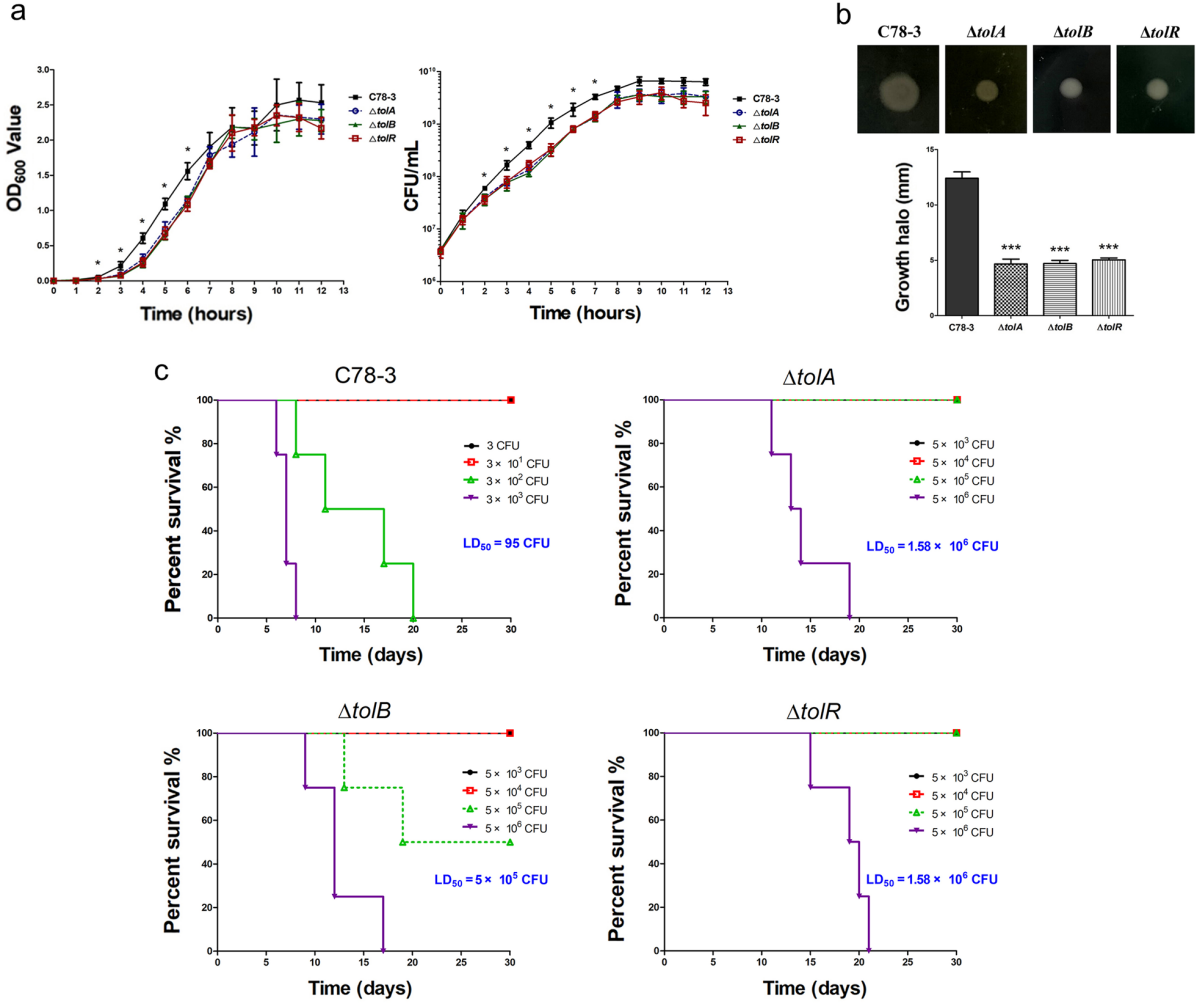
The roles of *tolA*, *tolB*, and *tolR* in cell growth, motility, and virulence of *S.* Choleraesuis. **a** Growth curves of C78-3, Δ*tolA*, Δ*tolB*, and Δ*tolR* using OD_600_ measurements, and the bacterial suspensions were serially diluted and plated to determine CFU numbers per milliliter. **b** Motility was evaluated by measuring the growth halo of C78-3, Δ*tolA*, Δ*tolB*, and Δ*tolR.* Each assay was completed in 3 independently conducted biology duplicates. *P < 0.05; **P < 0.01; ***P < 0.001. **c** LD_50_ evaluation of C78-3, Δ*tolA*, Δ*tolB*, and Δ*tolR* with a murine model. The infected mice were supervised every day for 30 days

To investigate the biological roles of *tolA*, *tolB*, and *tolR* in *S.* Choleraesuis, the growth curve, motility and virulence of the wild type C78-3 and mutants were studied. The growth of the Δ*tolA*, Δ*tolB*, and Δ*tolR* strains was significantly slower than C78-3 in the exponential phase, while there were no observed growth differences among the three mutants (Fig. 4a). The swimming halo diameter of C78-3, Δ*tolA*, Δ*tolB*, and Δ*tolR* was 12.4 mm, 4.7 mm, 4.7 mm, or 5.0 mm, respectively. These results indicated that the motility of *S.* Choleraesuis Δ*tolA*, Δ*tolB*, and Δ*tolR* was markedly impaired compared with the wild-type strain (Fig. 4b). We then examined the virulence of C78-3 and three mutants in a mouse model through the intraperitoneal route, the LD_50_ of C78-3, Δ*tolA*, Δ*tolB*, and Δ*tolR* were 95 CFU, 1.58 × 10^6^ CFU, 5 × 10^5^ CFU, or 1.58 × 10^6^ CFU, respectively (Fig. 4c). The LD_50_ value of three *S.* Choleraesuis mutants was significantly higher than that of C78-3. The results suggested that deletion of *tolA*, *tolB*, and *tolR* displayed an attenuated virulence of *S.* Choleraesuis in a mouse infection model.

### The involvement of *tolA*, *tolB*, and *tolR* in OMVs biogenesis of *S.* Choleraesuis

**Fig. 5:**
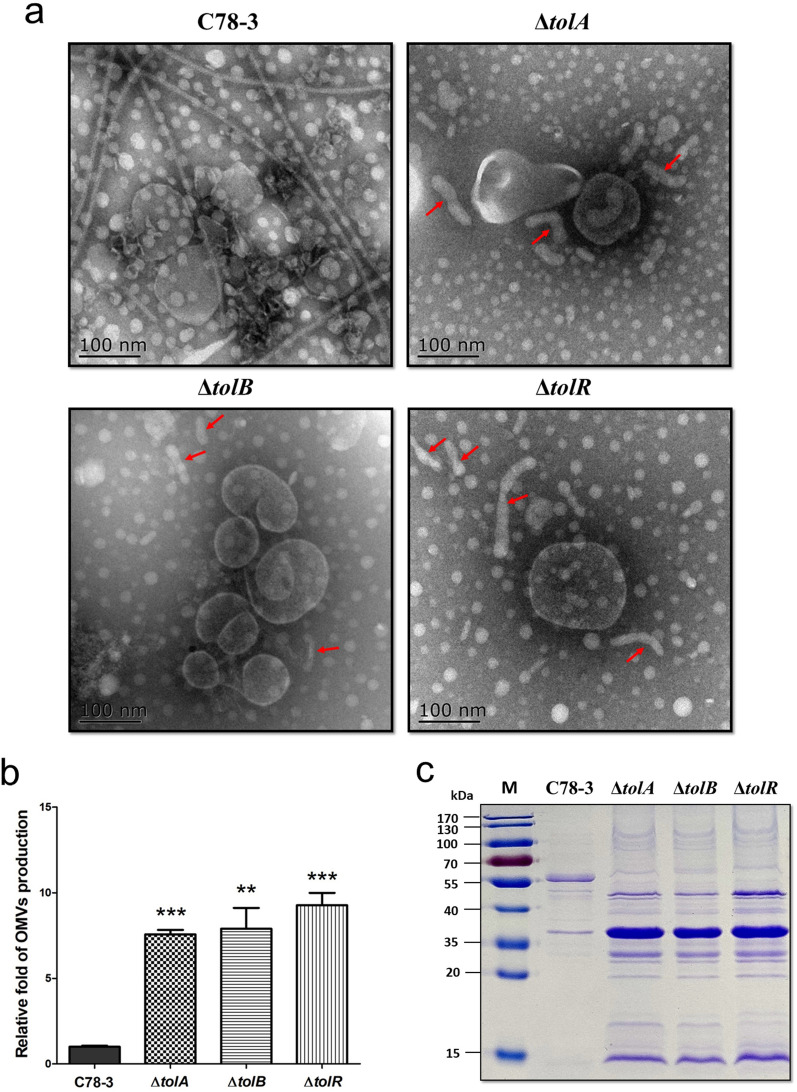
The involvement of *tolA*, *tolB*, and *tolR* in OMVs biogenesis of *S.* Choleraesuis. **a** OMVs derived from C78-3, Δ*tolA*, Δ*tolB*, and Δ*tolR* were evaluated by TEM. **b** OMVs production of C78-3 and its three mutants. The yield of OMVs was determined by quantitating the protein concentration. The assay was completed in 3 independently conducted biology duplicates. *P < 0.05; **P < 0.01; ***P < 0.001. **c** SDS-PAGE profile of OMVs derived from C78-3, Δ*tolA*, Δ*tolB*, and Δ*tolR* strains. Proteins were visualized using Coomassie Brilliant Blue R-250

At this point, we speculated that *S.* Choleraesuis *tolA*, *tolB*, and *tolR* genes participate in OMVs biogenesis for the following reasons: (1) all *S.* Choleraesuis *tolA*, *tolB*, and *tolR* mutants showed an impaired envelope integrity compared with the wild-type strain (Fig. 3); (2) vesicles could be clearly observed at the surface of *S.* Choleraesuis *tolA*, *tolB*, and *tolR* mutants (Fig. 2b). (3) according to previous reports, *tolA*, *tolB*, and *tolR* genes contribute to the OMVs biogenesis in *Salmonella enterica* serovars, like *tolA* and *tolB* of *S*. Typhimurium (Deatherage et al. 2009), and *tolR* of *S.* Typhi (Nevermann et al. 2019). To determine whether disruption of *tolA*, *tolB*, and *tolR* influence the OMVs biogenesis of *S.* Choleraesuis, we isolated OMVs from the wild-type and three mutants and further examined by TEM. As shown in Fig. 5a, all *S.* Choleraesuis mutants produced morphologically diverse OMVs regarding their shape (red arrow) and component (with or without flagella) compared with the wild-type strain. The OMVs of wild-type C78-3 has a large number of flagella. In contrast, no obvious flagella were observed in the OMVs preparations of the three mutants, which is consistent with the expressed non motile phenotype observed. In addition, the amount of OMVs in the Δ*tolA*, Δ*tolB*, and Δ*tolR* strains was higher than that in C78-3. To further test the OMVs yield of *S.* Choleraesuis C78-3 and its mutants, we determined the protein concentration of OMVs as an abundance indicator normalizing with CFU/ml according to a previous study (Deatherage et al. 2009). Our results showed that the Δ*tolA*, Δ*tolB*, and Δ*tolR* strains obviously exhibited more proteins in the OMVs fraction than the wild-type strain (Fig. 5b). Collectively, these data suggested that *tolA*, *tolB*, and *tolR* in *S.* Choleraesuis participate in the OMVs biogenesis.

A previous study demonstrated that OMVs cargo selection is closely related to the OMVs biogenesis (Schwechheimer and Kuehn 2015). Due to *tolA*, *tolB*, and *tolR* gene products are involved in the OMVs biogenesis, we speculated that OMVs cargo selection derived from *S*. Choleraesuis Δ*tolA*, Δ*tolB*, and Δ*tolR* strains should be influenced. To examine the differences in OMVs cargo selection, an SDS-PAGE was performed from the OMVs extracts. We observed that *S.* Choleraesuis wild-type OMVs presented few detectable proteins, with a major protein band at ~ 55 kDa (corresponding to flagellin) (Fig. 5c). The similar pattern of OMVs was also reported in a number of wild-type *Salmonella enterica* serovars, including *S*. Typhi (Nevermann et al. 2019), *S*. Typhimurium (Liu et al. 2016a), *S*. Choleraesuis (Liu et al. 2017b), and *S*. Enteritidis (Liu et al. 2017a). We found that the SDS-PAGE profiles of OMVs derived from *S*. Choleraesuis Δ*tolA*, Δ*tolB*, and Δ*tolR* strains (major protein bands at ~ 45 kDa, ~ 37 kDa, ~ 30 kDa, and ~ 15 kDa) were different from that of *S*. Choleraesuis wild-type OMVs, suggesting the involvement of *tolA*, *tolB*, and *tolR* genes in OMVs cargo selection.

Altogether, these results confirmed that *tolA*, *tolB*, and *tolR* genes participate in the OMVs biogenesis in *S.* Choleraesuis, increasing OMVs production, and affecting OMVs cargo selection.

### Evaluation of immune responses and protection against *S.* Choleraesuis

**Fig. 6:**
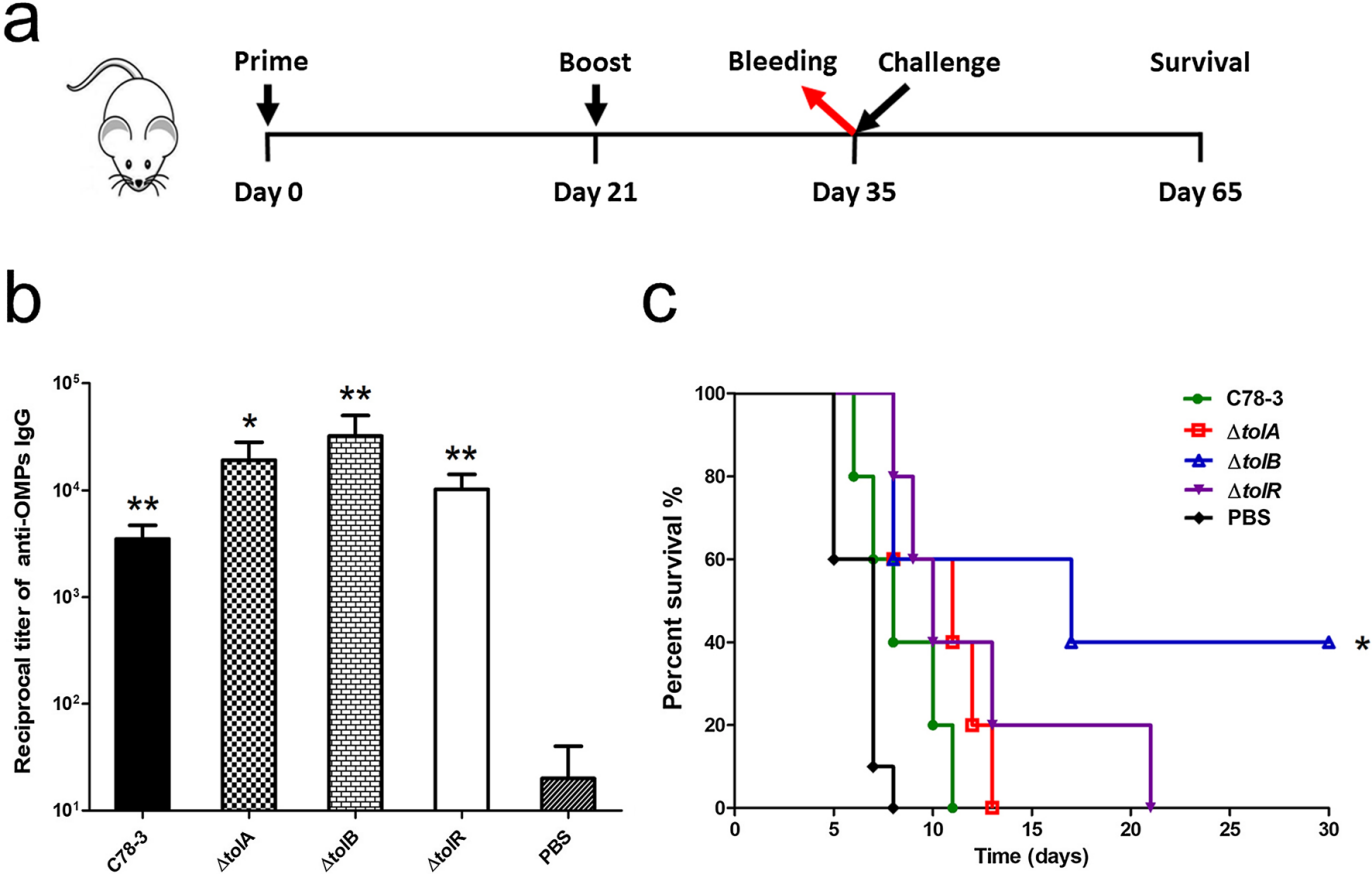
Evaluation of immune responses and protection against *S.* Choleraesuis. **a** Scheme of immunization regimen. **b** Antibody responses induced by OMVs derived from C78-3, Δ*tolA*, Δ*tolB*, and Δ*tolR* strains. IgG titers against OMPs from C78-3 and three mutants groups were remarkably higher in contrast to the PBS group. **c** Survival of the mice subjected to immunization with OMVs obtained from C78-3, Δ*tolA*, Δ*tolB*, and Δ*tolR* strains. Intraperitoneal immunizations of PBS served as the negative controls. Two weeks posterior to the reinforce immunizations, all animals were challenged with the wild-type *S.* Choleraesuis. The infected mice were supervised every day for 30 days. *P < 0.05; **P < 0.01; ***P < 0.001

To investigate the immune responses induced by OMVs, serum IgG was measured by ELISA against outer membrane proteins (OMPs) that were derived from *S*. Choleraesuis. Five groups of BALB/c mice were subjected to immunization for two times with OMVs or PBS via the intraperitoneal route (Fig. 6a). As shown in Fig. 6b, IgG titers against OMPs from C78-3 and three mutants groups were remarkably higher in contrast to the PBS group. Remarkably, although IgG titers against OMPs from Δ*tolA*, Δ*tolB*, and Δ*tolR* groups were slightly higher than the wild-type group, they had no significant difference. Two weeks after the booster immunizations, all animals were challenged with wild-type C78-3 via the oral route. All of the PBS control mice died within 8 days after C78-3 challenge, while immunization with OMVs of C78-3, Δ*tolA*, and Δ*tolR* prolonged mice survival to 11 days, 13 days, or 21 days, respectively (Fig. 6c). Specifically, immunization with Δ*tolB* OMVs conferred 40% protection to mice (Fig. 6c), which is significantly higher than that of the wild-type *S.* Choleraesuis OMVs. These data revealed that Δ*tolB* OMVs was able to provide partial protection against the wild-type *S*. Choleraesuis.

## Discussion

The *tol–pal* genes are not fully characterized in *S*. Choleraesuis, but they may play important roles in this pathogen considering that the multiple functions of this system in other bacteria. The present study aimed to characterize the function of *tolA*, *tolB*, and *tolR* genes in *S*. Choleraesuis. Indeed, *tol–pal* mutants have been studied extensively in many Gram-negative bacteria (Dennis et al. 1996; Dubuisson et al. 2005; Heilpern and Waldor 2000; Lahiri et al. 2011; Llamas et al. 2000; Prouty et al. 2002; Webster 1991). Mutations in *tol–pal* genes are impaired in their envelope integrity, affected cell morphology, increased sensitivity to bile salts, promoted OMVs production, reduced the cell growth, motility, and bacterial virulence.

Our analysis confirmed that *S*. Choleraesuis Δ*tolA*, Δ*tolB*, and Δ*tolR* display an altered cell morphology, which was supported by observing the microstructure of spherical forms, long chains, flagella defects, and membrane blebbing. A phenotype of altered morphology was also reported in *tol–pal* mutants of *S*. Typhimurium (Masilamani et al. 2018), *S*. Typhi (Lahiri et al. 2011), and *Erwinia chrysanthem* (Dubuisson et al. 2005). This phenomenon is mainly due to the disruption of envelope integrity. As previously observed, the Tol–Pal system mediated phosphatidylglycerols trafficking might affect envelope homeostasis that alters cell morphology (Masilamani et al. 2018). Previous studies have demonstrated that the Tol–Pal system of Gram-negative microbes is involved in maintaining OM integrity (Lazzaroni et al. 1999; Masilamani et al. 2018). According to our results, Δ*tolA*, Δ*tolB*, and Δ*tolR* mutants exhibited increased susceptibility to sodium deoxycholate and vancomycin, revealing that the envelope integrity might be damaged in these cases.

In *S*. Choleraesuis, deletion of *tolA*, *tolB*, or *tolR* is detrimental to the bacteria, since mutants grow very slowly in LB broth. In support of this, the lack of *tolA* and *tolB* in *Erwinia chrysanthemi* reduces cell growth (Dubuisson et al. 2005). Our data showed that *tolA*, *tolB*, and *tolR* genes were involved in motility of *S*. Choleraesuis. This phenotype was also observed in *tol–pal* mutants of *S*. Typhimurium (Nevermann et al. 2019), *E. coli* (Morgan et al. 2014), and *Erwinia chrysanthem* (Dubuisson et al. 2005). Our TEM observations provide a valuable explanation for this point. The *tolA*, *tolB*, and *tolR* mutations lack flagella, while the wild-type had a large number of flagella around the cell surface (Fig. 2b). By contrast, *S*. Typhi Δ*tolR* did not affect the motility (Nevermann et al. 2019), indicating that mutants lacking *tolR* in different *Salmonella enterica* serovars are not entirely equivalent.

*S*. Choleraesuis *tolA*, *tolB*, and *tolR* mutants displayed an attenuated virulence in a mouse infection model. Reduced virulence was also reported in *tol–pal* mutants of other pathogens. In *S*. Typhimurium, the virulence of *tolA*, *tolB*, and *tolR* mutants were significantly attenuated (Masilamani et al. 2018). In *E. coli*, the virulence of Δ*tolA* was largely attenuated using a *Galleria mellonella* model (Morgan et al. 2014). In *Erwinia chrysanthem*, expression of TolA or TolB was necessary for the full virulence in a potato tuber model (Dubuisson et al. 2005). The impaired virulence of *S*. Choleraesuis *tolA*, *tolB*, and *tolR* mutants probably results from the defective phenotypes, such as serious defects in cell morphology, envelope integrity, growth, and motility.

Thus far, the knowledge on the biogenesis of OMVs is still limited and fragmentary in *Salmonella* serotypes (Deatherage et al. 2009), and significantly lacking in *S*. Choleraesuis. Evidence has revealed that cross-linking between peptidoglycan and bacterial envelope proteins is an important mechanism for OMV biogenesis (Schwechheimer and Kuehn 2015). The Tol–Pal system corresponds to five envelope proteins that participates in OMVs biogenesis by interacting with peptidoglycan. The yield of OMVs increases when the cross-linking decreases. Accordingly, *S*. Typhimurium *tolA* and *tolB* mutants contribute to the production of OMVs (Deatherage et al. 2009). Moreover, the *tolR* mutation of *S*. Typhi displayed an increased production of OMVs (Nevermann et al. 2019). In *S*. Choleraesuis, we found that *tolA*, *tolB*, and *tolR* genes participate in the OMVs biogenesis. This study confirmed that TolA, TolB, and TolR are critical cell envelope proteins essential for OMV biogenesis.

Many studies have shown that native OMVs obtained from pathogens were able to confer strong protection against the challenge of pathogenic bacteria, such as *S*. Typhimurium (Liu et al. 2016a, b), *S*. Enteritidis (Liu et al. 2017a), *Shigella flexneri* (Camacho et al. 2011), *Acinetobacter baumannii* (McConnell et al. 2011), *Neisseria meningitidis* (Serruto et al. 2012). Remarkably, immunization with OMVs isolated from *S*. Choleraesuis wild type via the intraperitoneal route failed to confer protection against *S*. Choleraesuis. This result is likely due to the non-essential immune responses and excessive pro-inflammatory responses (McSorley et al. 2000; Singh et al. 2003; Smith et al. 2003), resulting in the failure of immune protection induced by OMVs. In support of this, similar results has been reported in a previous study (Liu et al. 2017b). Specifically, immunization with OMVs isolated from Δ*tolB* conferred 40% protection to mice. The result suggested that deletion of *tolB* in *S*. Choleraesuis can significantly improve the immunogenicity of OMVs, which is an intriguing discovery for OMVs of *S*. Choleraesuis, and the mechanism needs to be further evaluated.

This study confirmed that deletion of *S*. Choleraesuis *tolA*, *tolB*, and *tolR* genes severely damaged cell morphology, impaired the envelope integrity, inhibited growth and motility ability, and reduced the bacterial virulence. Moreover, *tolA*, *tolB*, and *tolR* genes also participate in the OMVs biogenesis, promoting OMVs production, and affecting OMVs cargo selection. In summary, this study provides an insight into the biological role of the *S.* Choleraesuis Tol–Pal system.

## Data Availability

Not applicable.
